# The impact of parental general anxiety disorder on parenting practices among Libyan parents: cross-sectional study

**DOI:** 10.1007/s44192-025-00156-y

**Published:** 2025-03-21

**Authors:** Basma Diaeddin Abuhadra, Rima Abohadra, Nobutoshi Nawa, Takeo Fujiwara

**Affiliations:** 1 Department of Public Health, Institute of Science Tokyo, Tokyo, Japan; 2 Pediatric Department, Faculty of Medicine, University of Tripoli, Tripoli, Libya

**Keywords:** Parental mental health, Anxiety, General anxiety disorder, Parenting practices, Positive parenting, Parental involvement, Poor supervision, Inconsistent discipline, Corporal punishment

## Abstract

**Background:**

Anxiety disorders is one of the most prevalent mental diseases globally, with cases rising by over 55% from 1990 to 2019. Recent research suggests anxiety can be contagious and may affect daily routines and parenting practices. In North African and Middle Eastern countries, where people face unique challenges such as natural disasters, war, and economic instability, the impact of anxiety on parenting is not well studied. This study aims to explore how general anxiety disorder (GAD) affects parenting styles and to estimate the prevalence and risk factors of GAD among Libyan parents, which are comparable to parents in the MENA region.

**Methods:**

Cross-sectional study was conducted in Libya, a MENA country, the sample included 233 parents aged 18–73 years who were assessed for anxiety and their parenting style by answering a self-administered online survey during the study period (1st May–18th October 2023), using (GAD-7) & (APQ) validated tools.

**Results:**

A total of 233 responses were analyzed. It was identified that anxious parents, in contrast to non-anxious parents adopted more poor supervision [$\beta$ 0.62, 95% CI (0.06–1.19)], corporal punishment [$\beta$ 0.86, 95% CI (0.18–1.55)] and less parental involvement practices [$\beta$ −0.8, 95% CI (−1.43 to −0.17)] after adjusting for age, sex, marital status, education, employment, family income, experiencing miscarriage, the number and sex of their children, and having a child with special needs. Additionally, the prevalence of GAD among Libyan parents was (48.93%). Sex [AOR 3.84, 95% CI (1.57–9.39)], family income [AOR 2.05, 95% CI (1.09–3.84)], and the number of children [AOR 3.23, CI (1.09–9.57)] were all significant predictors for anxiety.

**Conclusion:**

This study highlights the significant impact of parental GAD on parenting, showing trends like increased corporal punishment, poor supervision, and reduced involvement. These findings emphasize the need for targeted interventions to support anxious parents. Addressing parental mental health can improve family dynamics and break negative intergenerational cycles. Stakeholders and policymakers should prioritize mental health resources to foster positive parenting and mitigate the long-term effects of anxiety on children's development.

## Introduction

Child’s neurological, physiological, and psychosocial development and maturation are significantly affected by the parenting techniques their caregiver adopts, in particular during the initial stages of their lives. Childhood experiences during this period can either positively or negatively impact the child’s brain development and general well-being. Children raised in loving, supportive environments develop healthy attachments, trust, security, and self-esteem. In contrast, those exposed to abuse or neglect often feel unloved, struggle with trust, avoid close relationships, and perceive the world as threatening [1]. It is also worth noting that growing up in a dysfunctional home has negative consequences for children that may last into adulthood [2, 3].

Extensive research has focused on identifying the factors that influence a parent's ability to effectively supervise, and care for their children, which include child characteristics such as age, gender, physical and mental health, and disability [4, 5]. Also parent characteristics such as age, race [6], gender [4, 7], educational background [8, 9], mental health [6], and occupation [10, 11]. In addition, family socioeconomic status, the child’s ordinal position in the family [6, 9, 12], family history and life situation, cultural beliefs and way of child rearing or parenting [13], marital status [6, 7] and parental conflicts are major determinants.

Psychological difficulties in adults can impair their ability to function, leading to increased anger, reactivity, and social withdrawal, which may affect their parenting. Many adults with anxiety symptoms are also caregivers to children [14, 15].

Among the limited research that has investigated the potential consequences mental illness may have on parental functioning, a study indicated parents with social anxiety disorder, in particular, tend to be less warm, interpret their child's behavior more negatively, and correct them out of fear of looking bad [16]. Additionally, parents with GAD often struggle with emotion regulation, leading to inconsistent or inappropriate responses to their children. These challenges can contribute to adverse child mental health outcomes, including depression, anxiety [17], aggression, and rule-breaking behavior [18].

Few studies have explored this topic, with nearly all research conducted in developed Western countries. No studies have specifically examined the impact of parents' mental health on their parenting practices within the Arabic Middle Eastern and North African regions, further highlighting the significance of the present study, since it is the first attempt to investigate this topic within the region. A systematic review in 2021 identified the high prevalence of mental disorders in countries in the WHO’s Eastern Mediterranean Region (EMR). Depressive disorders showed the highest pooled prevalence of current, period, and lifetime prevalence (14.8%), followed by generalized anxiety disorder (GAD) (10.4%), though this is explainable since almost 85% of the EMR population has experienced a humanitarian crisis within the past two decades [19]. Libya is located in North Africa, however, it’s an Arab Muslim country that shares values, language, and religion with other Arab countries, and it serves as a great representation of Arabic MENA countries.

Based on a recent systematic review conducted in Libya, in 2023, it was identified that the prevalence of anxiety was 14.93%, indicating that this figure is alarmingly high compared to the global prevalence, and it explained that this elevation is probably triggered by all the challenges Libyans have endured [20]. The civil war in Libya has severely impacted mental health services, which were already inadequate before 2011, leaving some regions without any services. Due to stigma, families often avoid seeking professional help, turning first to spiritual healers or general practitioners, with psychiatrists considered a last resort [21].

Parenting is a lifelong process of nurturing children to become well-adjusted, independent adults, providing physical, emotional, social, and intellectual support. Over 75 years of research have identified three key parenting dimensions: parental support, behavioral control, and psychological control. Parental support fosters a strong emotional bond, leading to positive outcomes like reduced substance abuse [22]. Behavioral control, when balanced, is beneficial, but excessive control can harm development [23]. Psychological control is consistently linked to negative effects such as depression [24].

Parenting styles are classified into four types: authoritative (high demandingness and responsiveness), permissive, authoritarian, and neglectful. Authoritative parenting is associated with positive outcomes like psychosocial skills and academic success [25, 26]. Permissive parenting shows mixed results [26, 27], while authoritarian and neglectful parenting often leads to negative outcomes, including aggression, anxiety, and antisocial behavior [26, 27].

Parenting practices in Libya and the MENA region are deeply influenced by cultural and religious values, with Islamic teachings and traditional patriarchal structures playing a significant role. These cultural norms often dictate gender-specific expectations, where boys and girls are raised with different roles and responsibilities. The involvement of extended families is also prominent, reinforcing traditional gender roles. Socio-economic factors, such as the urban–rural divide and economic challenges, further shape parenting approaches, sometimes leading to more authoritarian practices. However, increasing education levels and exposure to global media are gradually introducing more modern, child-centered parenting styles, blending tradition with contemporary influences.

This study aims to investigate the effect parental GAD may have on parenting practices among Libyan parents and to examine the prevalence and risk factors of these symptoms among Libyan parents. As they represent a large percentage of the Libyan population.

## Material & methods

### Study design, setting, and period

An online cross-sectional survey was conducted using Google Forms among the Libyan population throughout the study period (1st May–18th October 2023). Data for this study were collected using a multi-sampling method (convenience, purposive, and snowballing techniques) by conveniently sharing the link of an online questionnaire through the most commonly used social media in Libya, and it was shared with friends, acquaintances, and networks (snowballing). Additionally, posters containing QR code directing to the survey were randomly, and purposefully distributed at a few schools in Libya, after obtaining approval from the Institutional Review Board approvals from Tokyo Medical and Dental University (Institute of Science Tokyo) (Approval number M2023-002).

The questionnaire was in Arabic language, and it consists of three sections. The first section consisted of questions on basic demographic characteristics and general questions related to socioeconomic status, educational background, age and sex of their children, it further inquired about any additional stressors, such as having a child with special needs and experiencing a miscarriage. The second section comprised the self-administered 7-item Generalized Anxiety Disorder Scale (GAD-7) with a cutoff score of $\ge$ 15, and the third section contained the self-administered 15-item Alabama Parenting Questionnaire. It is important to note that the questionnaire was conducted anonymously. Prior to participating in the survey, all participants provided informed consent.

Since mental health is a sensitive topic surrounded by stigma, especially in a conservative country like Libya, an anonymous online multi-sampling approach was adopted to ensure a sufficient sample was collected and that respondents would answer honestly. However, due to the online structure of the survey, it was not possible to estimate the response rate.

### Sample size

Based on the latest survey conducted by the Libyan Bureau of Census and Statistics in 2016, the number of persons above the age of 15 years old who are married, divorced, and widowed were 55.7% of the Libyan population [28].

The total sample size was calculated to be 103 respondents, using the formula SS = Z2*P* (1-P)/c2, where Z is the value from standard normal distribution corresponding to the desired confidence interval (Z = 1.96 for 95% CI), P is the estimated proportion (P = 55.7%), and c is the confidence interval (margin of error) (c = 9.6%).

### Inclusion & excluding criteria

The inclusion criteria were as follows: Libyan parents, who have at least one child above the age of 4 years and less than 18 years, who had lived for at least five consecutive years in Libya after the 2011 revolution, those who were able to read and write, and those aged 18 years or above. Responses from those who did not fully complete the questionnaire were excluded from the study.

### Measures

#### Generalized anxiety disorder scale (GAD-7)

The seven-item Generalized Anxiety Disorder scale (GAD-7) is used to detect anxiety symptoms. This tool includes questions regarding seven anxiety symptoms and their frequencies within the last 2 weeks. It has a specificity of 82% and a sensitivity of 89% [29], it has excellent internal consistency (Cronbach’s alpha = 0.83–0.93). Students rate the severity of symptoms on a 4-point Likert scale, resulting in a total score from 0 to 21, categorized as normal (0–4), mild (5–9), moderate (10–14), and severe (15–21). A score of 15 was regarded as the cutoff score to detect anxiety symptoms [29].

The Arabic version of the GAD-7, validated in Arabic samples with a Cronbach’s alpha of 0.92 [30], has also shown good internal consistency (Cronbach’s alpha = 0.88) in a pilot study in Libya [31].

#### Alabama parenting questionnaire (APQ)

The brief 15-item version was used, which assesses the five parenting subscales: Positive Parenting, Parental Involvement, Poor Supervision, Inconsistent Discipline, and Corporal Punishment, with each subscale being measured by three items, all items were scored using a 5-point scale. They used it in a sample of 208 children and adolescents ages 9–17, APQ-15 was robust across the child's age and gender, and it was able to distinguish between children with high and low conduct problems. Its validity was established by its correlation with observations of parenting behavior (38). APQ-15 was translated and adapted, and its psychometric properties were assessed for use in Arabic-speaking populations by Badahdah et al. [9].

### Statistical analysis

Descriptive statistical analyses of parent's and children's sociodemographic characteristics, if they experienced miscarriage, a child with special needs, parent GAD scores, and their parenting practices were examined in terms of frequency and percentage, as shown in (Table 1).

**Table 1:** Sociodemographic characteristics of parents

(N = 233)	Freq/Mean	Percent/SD
Anxiety		
Negative	119	51.07
Positive	114	48.93
Severity		
No anxiety	119	51.07
Mild anxiety	22	9.44
Moderate anxiety	49	21.03
Severe anxiety	43	18.45
Parenting style		
Positive parenting	13.11	2.21
Inconsistent discipline	9.14	2.56
Poor supervision	4.33	2.07
Parental involvement	11.39	2.38
Corporal punishment	6.48	2.53
Sociodemographic characteristic		
Parent sex		
Female	161	69.1
Male	72	30.9
Parent age		
18–25 years	4	1.75
26–35 years	79	34.65
36–45 years	93	40.79
46 years and above	52	22.81
Missing	5	2.15
Marital status		
Widow	6	2.58
Single	2	0.86
Married	216	92.7
Divorce	9	3.86
Level of education		
HighSchool	12	5.15
Graduate degree	88	37.77
Undergraduate/High inst	130	55.79
None of above	3	1.29
Employment status		
Non-Working	52	22.32
Working	181	77.68
Family income		
Less than 1000 LYD	33	14.16
1000 > 2500 LYD	101	43.35
More than 2500 LYD	99	42.49
Miscarriage		
Male	38	16.31
No	132	56.65
Yes	63	27.04
Children		
Number of children		
1 Child	33	14.16
Between 2 and 3	110	47.21
More than 3	90	38.63
Sex of children		
Females more	66	28.33
Males more	94	40.34
Equal male & female	70	30.04
Not specified	3	1.29
Special needs		
No	211	90.56
Yes	22	9.44

The association between parents with anxiety and their parenting behavior was estimated using multiple linear regression, as shown in (Tables 2, 3, 4, 5, 6) adjusted for potential confounders. The prevalence and risk factors of GAD among parents were also estimated by the use of multivariable logistic analysis, as shown in (Table 7).

**Table 2:** The association between parental Anxiety and Parental Involvement (N = 233)

	Parental involvement									
	Mean	SD	Crude model				Model 1			
			Coef	95% CI		p-value	Coef	95% CI		p-value
Anxiety										
No anxiety	11.64	2.39	Ref.				Ref.			
Positive anxiety	11.14	2.35	−0.50	−1.11	0.11	0.11	−0.80	−1.43	−0.17	**0.014**
Sex										
Female	11.79	2.00	Ref.							
Male	10.51	2.89	−1.27	−1.92	−0.63	**0**	−1.63	−2.54	−0.71	**0.001**
Parent age										
18–25 years	10.75	2.63	Ref.				Ref.			
26–35 years	11.33	2.48	0.58	−1.84	3.00	0.637	0.36	−2.21	2.93	0.784
36–45 years	11.59	2.28	0.84	−1.57	3.25	0.492	0.71	−1.93	3.35	0.595
46 years and above	11.19	2.44	0.44	−2.00	2.89	0.722	0.69	−2.03	3.40	0.619
Missing	11.40	2.30	0.65	−2.51	3.81	0.686	0.14	−3.26	3.55	0.934
Marital status										
Widow	13.17	1.33	Ref.				Ref.			
Single	13.00	2.83	−0.17	−3.98	3.64	0.931	1.60	−2.50	5.69	0.442
Married	11.31	2.38	−1.85	−3.78	0.08	0.06	−0.55	−2.59	1.49	0.596
Divorce	11.78	2.49	−1.39	−3.85	1.07	0.267	−0.32	−2.90	2.26	0.809
Education										
HighSchool	10.67	2.61	Ref.				Ref.			
Graduate degree	11.56	2.23	0.89	−0.54	2.32	0.222	0.67	−0.85	2.19	0.388
Undergraduate/High inst	11.28	2.44	0.62	−0.79	2.02	0.386	0.42	−1.06	1.90	0.577
None of above	14.33	1.15	3.67	0.66	6.67	**0.017**	4.27	1.19	7.36	**0.007**
Employment										
Non-working	11.04	2.27	Ref.				Ref.			
Working	11.50	2.41	0.46	−0.28	1.20	0.221	0.51	−0.24	1.27	0.18
Family income										
Less than 1000 LYD	11.61	2.40	Ref.				Ref.			
1000 > 2500 LYD	11.39	2.27	−0.22	−1.16	0.72	0.646	−0.04	−1.04	0.97	0.941
More than 2500 LYD	11.33	2.50	−0.27	−1.22	0.67	0.57	−0.28	−1.28	0.71	0.575
Miscarriage										
Male	10.45	2.97	Ref.				Ref.			
No	11.48	2.19	1.04	0.19	1.89	**0.017**	0.06	−0.97	1.09	0.909
Yes	11.78	2.24	1.33	0.38	2.28	**0.006**	0.16	−1.03	1.34	0.794
No of children										
1 child	10.76	3.19	Ref.				Ref.			
Between 2 and 3	11.58	2.24	0.82	−0.10	1.75	0.081	0.93	−0.08	1.93	0.07
More than 3	11.40	2.18	0.64	−0.31	1.59	0.185	0.73	−0.39	1.85	0.201
Sex of children										
Females more	11.08	2.46	Ref.				Ref.			
Males more	11.50	2.45	0.42	−0.33	1.18	0.269	0.47	−0.28	1.22	0.218
Equal male & female	11.51	2.22	0.44	−0.37	1.24	0.285	0.21	−0.62	1.05	0.618
Not specified	12.33	2.08	1.26	−1.51	4.03	0.372	0.79	−2.00	3.58	0.578
Special needs										
No	11.46	2.37	Ref.				Ref.			
Yes	10.73	2.41	−0.74	−1.78	0.31	0.167	−0.95	−2.01	0.10	0.076

*SD* Standard Deviation, *Coef.* Coefficient, *CI* Confidence interval

Model1- adjusted coefficient

Bold—Significant at p < 0.05

**Table 3:** The association between parental anxiety and positive parenting (N = 233)

	Positive parenting									
	Mean	SD	Crude model				Model 1			
Anxiety			Coef	95% CI		p-value	Coef	95% CI		p-value
No anxiety	13.27	2.16	Ref.				Ref.			
Positive anxiety	12.94	2.26	−0.33	−0.90	0.24	0.255	−0.44	−1.04	0.17	0.154
Sex										
Female	13.30	2.04	Ref.				Ref.			
Male	12.67	2.51	−0.64	−1.25	−0.02	**0.042**	−0.60	−1.48	0.28	0.178
Parent age										
18–25 years	14	1.41	Ref.				Ref.			
26–35 years	13.15	2.23	−0.85	−3.09	1.40	0.457	−1.54	−4.00	0.92	0.219
36–45 years	13.06	2.17	−0.94	−3.17	1.30	0.41	−1.61	−4.14	0.92	0.21
46 years and above	12.96	2.34	−1.04	−3.31	1.23	0.369	−1.72	−4.32	0.88	0.194
Missing	14.00	2.24	0.00	−2.94	2.94	1.00	−1.11	−4.37	2.15	0.504
Marital status										
Widow	14	1.10	Ref.				Ref.			
Single	13.50	2.12	−0.5	−4.04	3.04	0.781	−0.06	−3.98	3.86	0.976
Married	13.02	2.26	−0.98	−2.77	0.82	0.285	−0.86	−2.81	1.10	0.389
Divorce	14.44	0.73	0.44	−1.84	2.73	0.702	0.66	−1.81	3.14	0.597
Education										
HighSchool	12.25	2.49	Ref.				Ref.			
Graduate degree	13.24	2.30	0.99	−0.35	2.33	0.148	1.00	−0.45	2.46	0.176
Undergraduate/High inst	13.09	2.12	0.84	−0.47	2.16	0.209	0.80	−0.62	2.22	0.267
None of above	13.33	2.89	1.08	−1.73	3.90	0.449	1.65	−1.30	4.60	0.272
Employment										
Non-working	12.69	2.42	Ref.				Ref.			
Working	13.23	2.14	0.53	−0.15	1.22	0.125	0.36	−0.36	1.08	0.329
Family income										
Less than 1000 LYD	12.42	2.37	Ref.				Ref.			
1000 > 2500 LYD	13.22	2.26	0.79	−0.08	1.66	0.074	1.01	0.05	1.97	**0.04**
More than 2500 LYD	13.22	2.09	0.80	−0.07	1.67	0.073	0.91	−0.04	1.87	0.062
Miscarriage										
Male	12.47	2.76	Ref.				Ref.			
No	13.11	2.17	0.63	−0.16	1.43	0.119	0.14	−0.84	1.13	0.775
Yes	13.49	1.86	1.02	0.13	1.91	**0.025**	0.45	−0.69	1.59	0.436
No of children										
1 Child	13.0303	2.34	Ref.				Ref.			
Between 2 and 3	13.35	2.30	0.32	−0.55	1.18	0.473	0.61	−0.35	1.57	0.21
More than 3	12.84	2.04	−0.19	−1.07	0.70	0.68	0.35	−0.72	1.43	0.517
Sex of children										
Females more	13.39	1.98	Ref.				Ref.			
Males more	13.18	2.25	−0.21	−0.91	0.49	0.549	−0.15	−0.87	0.56	0.674
Equal male & female	12.73	2.38	−0.67	−1.41	0.08	0.081	−0.79	−1.59	0.01	0.052
Not specified	13.33	1.15	−0.06	−2.63	2.51	0.963	−0.38	−3.05	2.29	0.779
Special needs										
No	13.1896	2.08	Ref.				Ref.			
Yes	12.32	3.17	−0.87	−1.84	0.10	0.079	−0.88	−1.89	0.13	0.087

*SD* Standard Deviation, *Coef.* Coefficient, *CI* Confidence interval

Model1- adjusted coefficient

Bold—Significant at p < 0.05

**Table 4:** The association between parental anxiety and corporal punishment (N = 233)

	Corporal punishment									
	Mean	SD	Crude model				Model 1			
Anxiety			Coef	95% CI		p-value	Coef	95% CI		p-value
No anxiety	5.99	2.33	Ref.				Ref.			
Positive anxiety	6.99	2.64	1.00	0.36	1.64	**0.002**	0.86	0.18	1.55	**0.014**
Sex										
Female	6.65	2.58	Ref.							
Male	6.10	2.40	−0.55	−1.26	0.15	0.122	−0.43	−1.43	0.56	0.388
Parent age										
18–25 years	6.50	4.04	Ref.				Ref.			
26–35 years	6.90	2.57	0.40	−2.15	2.94	0.758	−0.06	−2.84	2.72	0.966
36–45 years	6.57	2.36	0.07	−2.47	2.61	0.957	−0.55	−3.41	2.30	0.702
46 years and above	5.88	2.62	−0.62	−3.19	1.96	0.638	−1.16	−4.11	1.78	0.436
Missing	4.40	1.67	−2.10	−5.41	1.21	0.213	−2.35	−6.04	1.33	0.209
Marital status										
Widow	6.67	3.56	Ref.				Ref.			
Single	5.00	2.83	−1.67	−5.75	2.41	0.422	−2.95	−7.38	1.48	0.191
Married	6.53	2.51	−0.13	−2.20	1.93	0.898	−0.03	−2.24	2.18	0.981
Divorce	5.44	2.40	−1.22	−3.86	1.41	0.362	−0.76	−3.55	2.04	0.594
Education										
HighSchool	7.42	2.78	Ref.				Ref.			
Graduate degree	6.53	2.63	−0.88	−2.42	0.66	0.259	−0.33	−1.97	1.32	0.696
Undergraduate/High inst	6.38	2.45	−1.04	−2.55	0.47	0.176	−0.58	−2.18	1.03	0.478
None of above	5.67	2.31	−1.75	−4.98	1.48	0.286	−1.23	−4.57	2.11	0.47
Employment										
Non-working	6.65	2.77	Ref.				Ref.			
Working	6.43	2.47	−0.22	−1.01	0.56	0.577	0.19	−0.63	1.01	0.649
Family income										
Less than 1000 LYD	7.21	2.81	Ref.				Ref.			
1000 > 2500 LYD	6.58	2.44	−0.63	−1.62	0.37	0.215	−0.77	−1.86	0.32	0.164
More than 2500 LYD	6.13	2.49	−1.08	−2.08	−0.08	**0.034**	−1.07	−2.15	0.01	0.053
Miscarriage										
Male	6.42	2.65	Ref.				Ref.			
No	6.73	2.59	0.31	−0.61	1.22	0.511	−0.31	−1.43	0.80	0.581
Yes	6.00	2.30	−0.42	−1.44	0.60	0.418	−1.15	−2.43	0.14	0.08
No of children										
1 Child	5.73	2.50	Ref.				Ref.			
Between 2 and 3	6.55	2.51	0.83	−0.16	1.81	0.1	1.10	0.02	2.18	0.046
More than 3	6.67	2.55	0.94	−0.07	1.95	0.069	1.56	0.35	2.77	0.012
Sex of children										
Females more	6.26	2.62	Ref.				Ref.			
Males more	6.62	2.56	0.36	−0.45	1.16	0.38	0.45	−0.36	1.26	0.276
Equal male & female	6.50	2.48	0.24	−0.62	1.10	0.579	−0.03	−0.94	0.87	0.947
Not specified	6.67	1.53	0.41	−2.55	3.37	0.786	0.48	−2.54	3.50	0.756
Special needs										
No	6.44	2.51	Ref.				Ref.			
Yes	6.91	2.74	0.47	−0.65	1.59	0.406	0.16	−0.98	1.30	0.784

*SD* Standard Deviation, *Coef.* Coefficient, *CI* Confidence interval

Model1- adjusted coefficient

Bold—Significant at p < 0.05

**Table 5:** The association between parental anxiety and poor supervision (N = 233)

	Poor supervision									
	Mean	SD	Crude Model				Model 1			
Anxiety			Coef	95% CI		p-value	Coef	95% CI		p-value
No anxiety	4.18	1.78	Ref.				Ref.			
Positive anxiety	4.49	2.33	0.31	−0.23	0.84	0.26	0.62	0.06	1.19	**0.03**
Sex										
Female	4.04	1.69	Ref.							
Male	5.00	2.64	0.18	−0.36	0.72	0.507	1.13	0.31	1.94	**0.007**
Parent age										
18–25 years	4.50	3	Ref.				Ref.			
26–35 years	3.96	1.60	−0.54	−2.64	1.56	0.615	0.65	−1.64	2.94	0.576
36–45 years	4.42	2.09	−0.08	−2.18	2.01	0.94	1.11	−1.24	3.46	0.354
46 years and above	4.73	2.59	0.23	−1.90	2.36	0.831	1.29	−1.14	3.71	0.296
Missing	4.40	1.14	−0.10	2.46	6.54	**0.00**	1.14	−1.89	4.18	0.458
Marital status										
Widow	4	1.26	Ref.				Ref.			
Single	6	4.24	2	−1.33	5.33	0.238	2.92	−0.72	6.57	0.116
Married	4.37	2.10	0.37	−1.32	2.06	0.666	0.44	−1.38	2.26	0.634
Divorce	3.33	0.71	−0.67	−2.82	1.48	0.542	−0.66	−2.96	1.64	0.572
Education										
HighSchool	4.58	2.43	Ref.				Ref.			
Graduate degree	4.47	2.20	−0.12	−1.38	1.14	0.855	−0.70	−2.06	0.65	0.306
Undergraduate/High inst	4.22	1.96	−0.36	−1.60	0.88	0.567	−0.76	−2.08	0.56	0.257
None of Above	4.33	2.31	−0.25	−2.90	2.40	0.853	−1.61	−4.36	1.14	0.251
Employment										
Non-working	4.17	1.90	Ref.				Ref.			
Working	4.38	2.12	0.21	−0.43	0.85	0.524	0.14	−0.53	0.81	0.686
Family income										
Less than 1000 LYD	4.27	2.34	Ref.							
1000 > 2500 LYD	4.10	1.66	−0.17	−0.99	0.64	0.676	−0.26	−1.15	0.64	0.569
More than 2500 LYD	4.60	2.33	0.32	−0.50	1.14	0.438	0.36	−0.53	1.25	0.427
Miscarriage										
Male	4.58	2.41	Ref.				Ref.			
No	4.48	2.17	−0.09	−0.84	0.65	0.804	0.92	0.00	1.84	0.049
Yes	3.87	1.52	−0.71	−1.54	0.13	0.097	0.49	−0.57	1.55	0.361
No of children										
1 child	4.73	2.55	Ref.				Ref.			
Between 2 and 3	4.07	1.65	−0.65	−1.46	0.15	0.112	−0.84	−1.73	0.06	0.066
More than 3	4.51	2.31	−0.22	−1.04	0.61	0.607	−0.61	−1.61	0.39	0.228
Sex of children										
Females more	4.14	1.81	Ref.				Ref.			
Males more	4.41	2.25	0.28	−0.38	0.94	0.404	0.22	−0.45	0.89	0.516
Equal male & female	4.47	2.10	0.34	−0.37	1.04	0.348	0.35	−0.40	1.09	0.36
Not specified	3	0	−1.14	−3.55	1.28	0.355	−0.75	−3.24	1.74	0.553
Special needs										
No	4.36	2.11	Ref.				Ref.			
Yes	4.09	1.74	−0.27	−1.19	0.65	0.563	−0.05	−0.98	0.89	0.925

*SD* Standard Deviation, *Coef.* Coefficient, *CI* Confidence interval

Model1- adjusted coefficient

Bold—Significant at p < 0.05

**Table 6:** The association between parental Anxiety and Inconsistent Discipline (N = 233)

	Inconsistent discipline									
	Mean	SD	Crude model				Model 1			
Anxiety			Coef	95% CI		p-value	Coef	95% CI		p-value
No anxiety	8.82	2.55	Ref.				Ref.			
Positive anxiety	9.48	2.53	0.67	0.01	1.32	**0.046**	0.70	−0.03	1.43	0.061
Sex										
Female	9.19	2.65	Ref.							
Male	9.03	2.36	−0.16	−0.88	0.55	0.651	−0.13	−1.19	0.93	0.804
Parent age										
18–25 years	9	3.37	Ref				Ref.			
26–35 years	9.14	2.99	0.14	−2.46	2.74	0.916	0.58	−2.40	3.55	0.704
36–45 years	9.25	2.25	0.25	−2.35	2.84	0.851	0.48	−2.58	3.54	0.758
46 years and above	9.10	2.37	0.10	−2.54	2.73	0.943	0.21	−2.94	3.36	0.897
Missing	7.80	2.39	−1.20	−4.60	2.20	0.488	−0.82	−4.77	3.12	0.682
Marital status										
Widow	9	1.79	Ref.				Ref.			
Single	10.50	0.71	1.5	−2.62	5.62	0.474	2.11	−2.63	6.86	0.381
Married	9.08	2.57	0.08	−2.01	2.17	0.937	0.17	−2.19	2.53	0.887
Divorce	10.33	2.83	1.33	−1.33	3.99	0.324	1.54	−1.46	4.53	0.313
Education										
HighSchool	9.42	3.00	Ref.				Ref.			
Graduate degree	9.26	2.50	−0.16	−1.72	1.40	0.845	0.10	−1.66	1.86	0.911
Undergraduate/High inst	9.05	2.59	−0.37	−1.90	1.16	0.634	−0.11	−1.83	1.61	0.898
None of above	8.67	2.08	−0.75	−4.02	2.52	0.652	−0.52	−4.10	3.05	0.773
Employment										
Non-working	9.04	2.75	Ref.				Ref.			
Working	9.17	2.51	0.13	−0.66	0.93	0.742	0.21	−0.67	1.08	0.641
Family income										
Less than 1000 LYD	9.12	2.87	Ref.				Ref.			
1000 > 2500 LYD	9.24	2.44	0.12	−0.90	1.13	0.821	0.15	−1.02	1.31	0.805
More than 2500 LYD	9.05	2.59	−0.07	−1.09	0.95	0.891	0.21	−0.95	1.37	0.72
Miscarriage										
Male	9.26	2.50	Ref.				Ref.			
No	9.17	2.40	−0.10	−1.03	0.84	0.839	−0.28	−1.48	0.91	0.639
Yes	9.02	2.93	−0.25	−1.29	0.79	0.64	−0.43	−1.80	0.95	0.54
No of children										
1 Child	9.18	3.16	Ref.				Ref.			
Between 2 and 3	9.03	2.67	−0.15	−1.16	0.85	0.762	0.08	−1.08	1.24	0.891
More than 3	9.27	2.18	0.08	−0.95	1.11	0.871	0.63	−0.67	1.93	0.339
Sex of children										
Females more	9	2.77	Ref.				Ref.			
Males more	9.33	2.47	0.33	−0.48	1.14	0.424	0.36	−0.51	1.23	0.412
Equal male & female	8.96	2.48	−0.04	−0.91	0.82	0.922	−0.21	−1.18	0.76	0.674
Not specified	10.67	2.89	1.67	−1.32	4.65	0.272	1.28	−1.96	4.51	0.437
Special needs										
No	9.12	2.55	Ref.				Ref.			
Yes	9.32	2.68	0.19	−0.94	1.33	0.735	−0.05	−1.28	1.17	0.93

*SD* Standard Deviation, *Coef.* Coefficient, *CI* Confidence interval

Model1- adjusted coefficient

Bold—Significant at p < 0.05

**Table 7:** The risk factors of anxiety (N = 233)

	Anxiety											
	Yes		No		Crude model				Model 1			
	Freq	%/SD	Freq	%/SD	OR	95% CI		p-value	OR	95% CI		p-value
Parent sex												
Female	93	81.58	68	57.14	3.32	1.83	6.03	**0.000**	3.84	1.57	9.39	**0.003**
Male	21	18.42	51	42.86	Ref.				Ref.			
Parent age												
18–25 years	4	3.51	0	0	Ref.				Ref.			
26–35 years	45	39.47	34	28.57	5.29	0.57	49.54	0.144	2.54	0.24	26.57	0.437
36–45 years	41	35.96	52	43.7	3.15	0.34	29.31	0.313	1.92	0.19	19.86	0.584
46 years and above	23	20.18	29	24.37	3.17	0.33	30.36	0.316	2.23	0.20	24.68	0.515
Missing	1	0.88	4	3.36	1.00				1.00			
Marital status												
Widow	4	3.51	2	1.68	2.15	0.39	12.01	0.381	1.37	0.20	9.49	0.75
Single	1	0.88	1	0.84	1.08	0.07	17.44	0.958	1.00			
Married	104	91.23	112	94.12	Ref.				Ref.			
Divorce	5	4.39	4	3.36	1.35	0.35	5.15	0.664	0.47	0.10	2.27	0.344
Education												
HighSchool	7	6.14	5	4.2	Ref.				Ref.			
Graduate degree	43	37.72	45	37.82	0.68	0.20	2.32	0.54	1.09	0.25	4.78	0.905
Undergraduate/High inst	62	54.39	68	57.14	0.65	0.20	2.16	0.483	0.75	0.18	3.20	0.701
None of Above	2	1.75	1	0.84	1.43	0.10	20.44	0.793	1.30	0.06	26.96	0.867
Employment												
Non-working	30	26.32	22	18.49	1.57	0.84	2.94	0.153	1.29	0.63	2.64	0.482
Working	84	73.68	97	81.51	Ref.				Ref.			
Family income												
Less than 1000 LYD	19	16.67	14	11.76	2.09	0.94	4.64	0.071	1.44	0.57	3.68	0.441
1000 > 2500 LYD	56	49.12	45	37.82	1.91	1.09	3.36	**0.024**	2.05	1.09	3.84	**0.025**
More than 2500 LYD	39	34.21	60	50.42	Ref.				Ref.			
Miscarriage												
Male	12	10.53	26	21.85	0.48	0.2	1.11	0.085	1.60	0.5	5.15	0.427
No	71	62.28	61	51.26	1.20	0.66	2.19	0.549	1.46	0.74	2.85	0.274
Yes	31	27.19	32	26.89	Ref.				Ref.			
No of children												
1 Child	22	19.3	11	9.24	2.50	1.09	5.76	**0.031**	3.23	1.09	9.57	**0.034**
Between 2 and 3	52	45.61	58	48.74	1.12	0.64	1.96	0.69	1.21	0.61	2.41	0.581
More than 3	40	35.09	50	42.02	Ref.				Ref.			
Sex of children												
Females more	32	28.07	34	28.57	1.02	0.55	1.92	0.939	0.91	0.45	1.87	0.806
Males more	45	39.47	49	41.18	Ref.				Ref.			
Equal male & female	35	30.7	35	29.41	1.09	0.59	2.02	0.787	1.17	0.57	2.40	0.672
Not specified	2	1.75	1	0.84	2.18	0.19	24.84	0.531	1.61	0.12	22.34	0.722
Special needs												
No	102	89.47	109	91.6	Ref.				Ref.			
Yes	12	10.53	10	8.4	1.28	0.53	3.10	0.58	1.57	0.59	4.18	0.362

*OR* Odd ratio, *CI* Confidence interval

Model 1- adjusted Ors

Bold-Significant at p < 0.05

Statistical analyses were performed using Stata Statistical software version 17.0 for Macintosh, the significance level was set at 0.05.

## Results

Of the 261 persons who answered to the questionnaire, 233 met the inclusion criteria. Given that 6 participants did not provide consent, 7 were excluded considering they were non-Libyans, 13 were excluded because they did not reside in Libya for more than 5 years after the revolution, and two others were deemed invalid since one was 17 years old and the other had no children.

### Demographic characteristics of sample

Of the 261 parents who responded, while 28 entries were considered invalid, the remaining 233 valid samples consisted of (69.1% mothers and 30.9% fathers) with ages ranging between 22 and 73 years (Mean age 39.72, SD = 8.9), with approximately 75% were among the age group 26–45. Almost all the parents were married (92.7%), with nine divorced (all females), six widowed (all females), and two single (one male and one female). the largest number of parents (47.2%) had between two and three children, followed by those who had more than three children (38.6%), and single child (14.16%). Almost two-third of the parents (62.23%) had children of both genders, while (23.18%) had only boys, and the remaining (13.3%) had only girls. Furthermore, (40.34%) of parents indicated they had more boys, (28.33%) indicated they had more girls, while (30.04%) of parents indicated having an equal number of both boys and girls.

Almost a quarter of the parents (22.32%) were not working at the time of the data collection (12.88% unemployed, 6.87% students, and 2.58% retired), while the remaining three quarters (77.68%) were working, almost all of them were employees (60.9%), while (6.44%) identified themselves as self-employed and (10.3%) didn’t specify their work.

The largest number of parents (43.25%) indicated that their total monthly income was between (1000–2500 LYD), followed by (42.49%) indicated that their total monthly income was more than 2500 LYD, and the remaining (14.16%) indicated that they had an income of less than 1000 LYD. More than half of the parents (55.79%) had a graduate degree or higher, followed by (37.77%) parents who had an undergraduate / high institute degree, and the remaining (5.15%) had a high school diploma. More than a quarter of the parents (27.04%) have experienced the pain of miscarriage, and (9.44%) parents had a child with special needs, more than half of these parents had ADHD, as shown in (Table 1).

### The association between parental anxiety and parenting practices

#### Positive parenting practices

Positive parenting and parental involvement practices were less prevalent among parents identified with anxiety in contrast with non-anxious parents, this association was statistically significant for the parental involvement domain after adjusting for the identified confounders [P.I $\beta$ −0.8, 95% CI (−1.43 to −0.17), p-value 0.014] as shown in (Table 2), while positive parenting wasn’t statistically significant [P.P $\beta$ −0.44, 95% CI (−1.04 to 0.17), p-value 0.154] as shown in (Table 3).

#### Negative parenting practices

Corporal punishment and poor supervision practices were expressed to a greater extent in parents identified with anxiety symptoms, in contrast to non-anxious. Its association with the corporal punishment domain was statistically significant [C.P $\beta$ 1.0, 95% CI (0.30–1.64), p-value 0.002] and remained significant even after controlling for all the identified confounders [C.P $\beta$ 0.86, 95% CI (0.18–1.55), p-value 0.014] as shown in (Table 4). However, its association with the poor supervision domain became statistically significant after adjusting for all the identified confounders [P.S $\beta$ 0.62, 95% CI (0.06–1.19), p-value 0.03] as shown in (Table 5).

Anxious parents adopted more inconsistency in their disciplinary measures towards their children compared to not-anxious parents, which was statistically significant [I.D $\beta$ 0.67, 95% CI (0.01–1.32), p-value 0.046], though it’s no longer significant after controlling for all the identified variables [I.D $\beta$ 0.7, 95% CI (−0.03 to 1.43), p-value 0.061] as shown in (Table 6).

In conclusion, parents who identified with anxiety symptoms, in contrast to non-anxious parents adopted more poor supervision and corporal punishment practices and showed less involvement in their children’s lives.

### Prevalence and risk factors of anxiety among parents

One hundred and fourteen individuals exhibited anxiety symptoms among the two hundred and thirty-three individuals who participated in this study, utilizing the GAD-7 with a cutoff score of $\ge$ 15, indicating a prevalence of 48.93% (95% confidence interval: 42.5–55.4%) among this study sample. Further confirming the severity of anxiety disorder in the Libyan population, this study sample indicated that more than three quarters (78.68%) of those identified as anxious parents were either recognized to be among the moderate (42.98%), and severe anxiety (37.7%) groups, thus necessitating immediate attention. The remaining of individuals exhibited mild symptoms (19.29%).

We utilized a multiple logistic regression methodology to ascertain the risk factors associated with anxiety, as demonstrated in (Table (7). The analysis revealed that females were 3.84 times more likely to develop anxiety compared to males [AOR 3.84, 95% CI (1.57–9.39), p-value 0.030]. Parents with a moderate income (1000 > 2500 LYD) were twice as likely to develop anxiety [AOR 2.05, 95% CI (1.09–3.84), p-value 0.025]. Conversely, parents with a low income (less than 1000 LYD) demonstrated a 1.44 times higher likelihood of experiencing anxiety compared to those with a high income (more than 2500 LYD) [AOR 1.44, 95% CI (0.57–3.68), p-value 0.441]. Furthermore, parents with a single child were 3.23 times more likely to experience anxiety than those with more than three children [AOR 3.23, 95% CI (1.09–9.57), p-value 0.034].

This study found strong associations between parental gender, family income, and the number of children with the development of anxiety symptoms, as shown in (Table 7).

## Discussion

This study aimed to investigate the effect parental anxiety may have on parenting practices, by exploring the most adopted parental practices among those identified with anxiety and those without. Five main parenting behaviors were measured; three negative behaviors (Inconsistent Discipline, Poor Supervision, and Corporal Punishment), two positive behaviors (Positive parenting and Parental Involvement) and later assessed if these results were statistically significant.

Anxious parents within this study, in contrast to non-anxious parents, presented a significant positive association with poor supervision, and corporal punishment practices and showed a significant negative correlation with parental involvement in their children’s lives. There is little recent available information on the parenting characteristics of parents with anxiety disorders, despite the fact that investigations focusing on high-risk populations have revealed a substantial prevalence of anxiety disorders in the offspring of anxious parents, which cannot be solely attributed to genetic inheritance [32]. As most studies within this field addressed anxiety-promoting parenting behaviors usually adopted by anxious parents (such as overcontrol and low parental warmth, criticism, granting of autonomy, hostility, and anxious modeling) in an attempt to understand the transitional pathway of anxiety, none of them addressed the five-parenting behaviors that were measured within this study. However, a certain study by (Chilcoat et al.) has identified that anxiety-disordered mothers have monitored their children less than other mothers [33] confirming the findings of this research. Another study indicated parents with social anxiety disorder showed less warmth and less positive affect [16], which logically could be attributed to poor involvement, and negatively affect parental involvement, consistent with the results of this study. A possible explanation, anxious parents may struggle to provide emotional support and find it challenging to engage in quality time with their children, limiting parents’ involvement with the child. Additionally, they struggle with persistent worry and preoccupation, which can lead to difficulties in maintaining focused and attentive supervision. As for corporal punishment, anxiety can impact a parent's emotional regulation, and become more prone to stress, potentially leading to impulsive or reactive behaviors such as corporal punishment.

In regard to the prevalence and risk factors of anxiety, the study identified that parents had an alarmingly high prevalence of 49%, which cannot be further from the truth, considering the range of prevalence reported among the eighteen studies conducted in Libya (5.6–91.5%), while most of them reported a prevalence between (45–65%) [20]. Furthermore, the prevalence of anxiety among this study participants is also alarmingly high compared to the global prevalence among conflict-affected countries (21.7%) [34] and the aggregate prevalence reported for anxiety in Libya (14.93%) [20]. Moreover, anxiety had a significant association with sex, income, and number of children. Women were 3.84 times more likely compared to men, low-income families were 1.44 times more likely compared to high-income families, and parents with one child were 3.23 times more likely, compared to those who had more than 3 children, to develop anxiety for all. It was also identified that anxiety is more prevalent among older, divorced, widowed, and non-working individuals, and who have children with special needs. Similar to a recent study in Libya, that presented a similar association for sex, education, marital status, and working status, with the exception of age, which had a contrasting association [35]. Furthermore, other studies conducted within low and middle-income countries (Latin America, India and China) endorsed the association of anxiety with age, sex, and social economic status [36].

While the correlation with parents of children with special needs in this study doesn’t exhibit statistical significance, numerous studies have affirmed this positive association. For instance, a study conducted in Libya examined this relationship among parents of children diagnosed with autism [37], whereas another study explored it among parents of children diagnosed with both autism and Down syndrome [38]. Furthermore, in Japan, it was examined among parents of children diagnosed with pervasive developmental disorders (PDD) [39]. All of these studies provide evidence of an elevated level of anxiety and stress experienced by parents, as they were most probably the primary caregivers of their children and thus had less time for themselves since most of their time was mostly spent fulfilling their children’s needs. Additionally, these findings can be explained by the sense of frustration that arises from having a disabled or behaviorally challenged child, feelings of guilt stemming from anger and rejection towards the child, the social stigma associated with the disorder, and also the additional financial expenses endured by these parents, contributing to a financial burden.

Despite the fact that there is a lack of consensus in the literature regarding the negative correlation found in this study between anxiety and the number of children that parents may have, it can be logically explained that parents with larger families may have more available support to help them navigate stressful challenges, while having more children may increase parenting stress due to the additional demands and responsibilities. However, it does not necessarily imply that parents will experience higher levels of stress, and anxiety. Some parents may find fulfillment and joy in raising multiple children, which could serve as a protective factor (buffer). Additionally, parents with more children may have developed certain personality traits and coping strategies to navigate stressful situations. Further research is required to gain a better understanding of the complex interplay between the aforementioned mental illness and family size, as well as to identify strategies for supporting parental mental health across different family contexts.

## Strengths and limitations

### Strengths

One of the greatest strengths of this study is that it fills a critical research gap in the Middle Eastern and North African region, as it is the first to address the association between anxiety and parenting styles, a topic previously studied in other parts of the world but not in this region. This is of great importance given the unique cultural and social challenges in this area. This study also has several other strengths. For instance, validated and psychometrically sound measures of anxiety and parenting behaviors have been employed in this study. Additionally, various demographic variables for both parent and child, as well as other specific variables such as having a child with special needs or experiencing miscarriage, which have been found to be connected to both mental well-being and parenting behavior in certain studies, were controlled for in the statistical analyses.

### Limitations

The findings of this study should be interpreted with certain methodological limitations in mind. Firstly, the reliance on self-report measures for parenting hindered our ability to collect data from multiple sources, such as spouses and children, and to employ multi-method data like clinical interviews and observational measures. Individuals might have difficulties accurately identifying their own emotional states, potentially misrepresenting the frequency and intensity of their symptoms and emotions. This limitation implies that the observed associations might partly stem from the influence of anxiety on perceptions of parenting rather than actual parenting practices. Additionally, mothers concerned with impression management might have underreported negative behaviors, minimizing parenting challenges due to societal pressures.

Although current findings show an association between parental mental disorders and parenting practices, they were measured cross-sectionally, preventing conclusions about causation or the directionality of effects. Parenting-related pressures might intensify symptoms of anxiety, and these relationships could be reciprocal and transactional. More complex empirical models are needed to account for these linkages. Another limitation was the use of an online survey, excluding those without internet access. Misleading responses from male participants to the question about experiencing a miscarriage further impacted the results. Finally, resource limitations prevented us from collecting data on child behavior, prior medical and mental health, type of trauma, cultural values, social pressure, and social support, which are important factors associated with parenting and mental health. Despite these limitations, the novel results that emerged bear replication.

## Future directions and clinical implications

Further research within this field should be broader in design and methodology, incorporating longitudinal research designs to capture developmental processes and changes in parenting over time, particularly following stressful events. Studying families with parents experiencing long-term anxiety from a resilience perspective can offer insights into resilience pathways and other protective factors. Expanding the methodological palette to include dynamic assessments like Ecological Momentary Assessment (EMA) or observational measurements, alongside children's reports, can enhance our understanding of the interplay between mental disorders and parenting behavior, addressing the current lack of research in this area.

The evidence indicates that parental trauma history is linked to significant parenting challenges and adverse child outcomes, yet interventions addressing these issues are underdeveloped. There is a need for empirically grounded parenting skills programs targeting trauma-related difficulties, such as emotion regulation and conflict management. Adopting effective preventive interventions for families impacted by crises can enhance parenting practices, improve parent–child relationships, and increase social support. Randomized prevention trials have shown benefits in parents' mental health, parenting skills, and child adjustment [40]. Healthcare providers should educate parents with mental illness about negative parenting outcomes and refer them to preventive programs, emphasizing stress management techniques to help maintain consistency in parenting strategies.

## Conclusion

The findings of this study highlight the profound impact of parental anxiety on parenting practices, revealing concerning trends such as increased corporal punishment, poor supervision, and reduced parental involvement. These results underscore the urgent need for targeted interventions aimed at supporting parents grappling with anxiety symptoms. By addressing the mental health needs of parents, we have the potential to disrupt the cycle of negative intergenerational outcomes, foster healthier family dynamics, and promote the well-being of future generations. This research holds particular significance for Libya and its neighboring MENA countries, offering valuable insights that can inform tailored strategies for psychological support and intervention within these communities. It is imperative that stakeholders and policymakers prioritize resources towards addressing parental mental health as a means of promoting positive parenting practices and mitigating the long-term consequences of anxiety on children's development.

## Data Availability

The datasets used and/or analyzed during the current study are available from the corresponding author on reasonable request.
